# Non-fasting High-Density Lipoprotein Is Associated With White Matter Microstructure in Healthy Older Adults

**DOI:** 10.3389/fnagi.2019.00100

**Published:** 2019-05-07

**Authors:** Nathan F. Johnson, Brian T. Gold, Dorothy Ross, Alison L. Bailey, Jody L. Clasey, Vedant Gupta, Steve W. Leung, David K. Powell

**Affiliations:** ^1^Department of Rehabilitation Sciences, Division of Physical Therapy, University of Kentucky, Lexington, KY, United States; ^2^Neuroscience Department, University of Kentucky, Lexington, KY, United States; ^3^Magnetic Resonance Imaging and Spectroscopy Center, University of Kentucky, Lexington, KY, United States; ^4^Sanders-Brown Center on Aging, University of Kentucky, Lexington, KY, United States; ^5^Clinical Services Core, University of Kentucky, Lexington, KY, United States; ^6^Erlanger Heart and Lung Institute, University of Tennessee College of Medicine Chattanooga, Chattanooga, TN, United States; ^7^Department of Kinesiology and Health Promotion, University of Kentucky, Lexington, KY, United States; ^8^Gill Heart and Vascular Institute, University of Kentucky, Lexington, KY, United States

**Keywords:** high-density lipoprotein, white matter, fornix, cholesterol, aging

## Abstract

A growing body of evidence indicates that biomarkers of cardiovascular risk may be related to cerebral health. However, little is known about the role that non-fasting lipoproteins play in assessing age-related declines in a cerebral biomarker sensitive to vascular compromise, white matter (WM) microstructure. High-density lipoprotein cholesterol (HDL-C) is atheroprotective and low-density lipoprotein cholesterol (LDL-C) is a major atherogenic lipoprotein. This study explored the relationships between non-fasting levels of cholesterol and WM microstructure in healthy older adults. A voxelwise and region of interest approach was used to determine the relationship between cholesterol and fractional anisotropy (FA). Participants included 87 older adults between the ages of 59 and 77 (mean age = 65.5 years, SD = 3.9). Results indicated that higher HDL-C was associated with higher FA in diffuse regions of the brain when controlling for age, sex, and body mass index (BMI). HDL-C was also positively associated with FA in the corpus callosum and fornix. No relationship was observed between LDL-C and FA. Findings suggest that a modifiable lifestyle variable associated with cardiovascular health may help to preserve cerebral WM.

## Introduction

Elevated levels of cholesterol are linked to atherosclerotic and cerebrovascular disease (Yaghi and Elkind, 2015). Hypercholesterolemia, high levels of low-density lipoprotein cholesterol (LDL-C), and dyslipidemia, low levels of high-density lipoprotein cholesterol (HDL-C), are associated with a number of neurological pathologies (Anstey et al., 2008; Crisby et al., 2010; Ward et al., 2010; Segatto et al., 2014). LDL-C is considered to be the major atherogenic lipoprotein (Young and Parthasarathy, 1994). HDL-C is known to play a role in reverse transport of free cholesterol (Genest et al., 1999; Von Eckardstein and Assmann, 2000), and is atheroprotective (Stein and Stein, 1999). A plethora of research demonstrates that proper regulation of lipoproteins preserves vascular health. However, less is known about the relationship between cholesterol and neuroimaging measures of neuronal health, or white matter (WM) microstructure.

Divergent associations have been reported between serum lipoprotein levels and WM microstructure in healthy adults (Williams et al., 2013; Lou et al., 2014; Warstadt et al., 2014). Inverse relationships between fasting LDL-C and WM microstructure (fractional anisotropy; FA) have been reported in young adults (Lou et al., 2014) and in a sample of middle-aged and older adults (Williams et al., 2013). An absence of a relationship has also been reported when using non-fasting adolescent LDL-C values to predict young adulthood FA values (Warstadt et al., 2014). However, Warstadt et al. (2014) did observe a relationship between non-fasting adolescent HDL-C and FA in young adulthood. Finally, Williams et al. (2013) report both positive and negative relationships between FA and fasting HDL-C in a sample of middle-aged and older adults. Discrepant findings are likely due to heterogeneous age groups, across and within sample populations, differences in neuroimaging methodology, cultural and lifestyle variables, and a collection of fasting and non-fasting lipid levels.

Recent evidence supports the use of non-fasting lipid biomarkers when assessing cardiovascular risk (Driver et al., 2016; Mora, 2016; Nordestgaard et al., 2016a,b). Non-fasting HDL-C levels may be more clinically relevant as they best approximate the atheroprotective benefits of lifestyle behaviors. Low levels of HDL-C are associated with deleterious changes in cognition, gray matter (GM) and WM (Crisby et al., 2010; Ward et al., 2010). HDL-C also helps to prevent the deleterious effects of β-amyloid (Aβ) deposition (Koudinov et al., 2001; Robert et al., 2017; Chernick et al., 2018). Thus, understanding the relationship between HDL-C and brain structure will provide a better understanding of how an atheroprotective modifiable risk factor attenuates age-related declines in WM microstructure.

WM microstructure declines with age (Madden et al., 2004; Johnson et al., 2013), and non-modifiable variables, such as APOEε4+ carrier status, have detrimental effects on WM microstructure. The corpus callosum, cingulum, inferior longitudinal fasciculus (ILF), and internal capsule represent WM tracts sensitive to APOEε4+ carrier status (Cavedo et al., 2017) and demonstrate declines in early Alzheimer’s disease (AD) pathology (Xie et al., 2006; Sydykova et al., 2007; Teipel et al., 2007; Tsao et al., 2014; Lee et al., 2016). Significant gaps remain in the literature regarding the relationship between non-modifiable variables, such as APOE status, and modifiable lifestyle variables, such as cholesterol (Anstey et al., 2017). This study focuses on the relationship between a modifiable lifestyle variable, cholesterol, and WM microstructure across these sensitive regions.

Another WM tract that is sensitive to aging and neurodegeneration is the fornix (Stadlbauer et al., 2008b; Yasmin et al., 2009; Michielse et al., 2010; Sullivan et al., 2010). The fornix is a bi-directional pathway to, from, and between hippocampi. It is comprised of cholinergic inputs, and a diversity of efferent pathways to subcortical and prefrontal regions. The fornix plays a critical role in episodic memory (Douet and Chang, 2015). Fornix microstructure is associated with memory performance (Rudebeck et al., 2009; Zahr et al., 2009; Fletcher et al., 2013), and represents a non-invasive biomarker of preclinical AD (Nowrangi and Rosenberg, 2015). Thus, the fornix also warrants selective attention in determining the protective or deleterious effects of serum lipoproteins on WM microstructure.

In the present study, we used diffusion tensor imaging (DTI) to determine the relationship between serum cholesterol and WM microstructure. HDL-C was of primary interest due to its long-standing predictability of cardiovascular disease (Rahman et al., 2018), but WM-LDL-C relationships were also considered. Specifically, we explored potential relationships between HDL-C, LDL-C and WM microstructure using a voxelwise and a region of interest approach. WM regions that were previously shown to be sensitive to aging, carrier status and AD were of primary interest (Madden et al., 2004; Stadlbauer et al., 2008a; Michielse et al., 2010; Cavedo et al., 2017).

## Materials and Methods

### Participants

A total of 87 (34 males; mean age = 65.5, SD = 3.9) right-handed subjects were included in this analysis. Participant data were combined across two different neuroimaging studies at the University of Kentucky. Informed consent was obtained from each participant under an approved University of Kentucky Institutional Review Board protocol. Participants met all criteria for participating in a magnetic resonance imaging (MRI) study. Exclusion for the MRI study included history of a major head injury and/or concussion, neurological disorder (e.g., stroke, seizure), a major psychiatric condition (e.g., depression), uncontrolled hypertension or diabetes mellitus, reported psychotropic drug use, or the presence of metal fragments and/or metallic implants that could cause bodily injury or disrupt the magnetic field. Mini Mental State Examination (MMSE) scores determined that all participants were cognitively healthy at the time of testing (mean = 29.0, SD = 1.1, minimum = 25).

### Blood Draws

Venipuncture was used to collect all non-fasting blood samples. Samples were collected from the antecubital area of the arm. Samples were placed into four vacuum tubes. Two sample tubes (3.0 mL with Lithium Heparin, and 3.0 mL with EDTA) were sent to UK Healthcare Clinical Laboratory for Basic Metabolic Panel, Lipid Panel, and Hematocrit. Lipid profile analyses were performed by the University of Kentucky Center for Clinical and Translational Science Biomarker Analytics Lab. The Roche Cobas 8000 analyzer (Roche Diagnostics, Mannheim, Germany) was used to determine cholesterol levels.

### Diffusion Tensor Imaging Acquisition

Data were acquired on a 3T TIM Siemens scanner at the University of Kentucky’s MRI and Spectroscopy Center. A 32-channel head coil was used. Whole brain diffusion tensor images were acquired with 60 non-collinear encoding directions (*b* = 1,000 s/mm^2^) and eight images without diffusion weighting (*b* = 0 s/mm^2^, b0), using an axial double refocused spin echo EPI sequence (TR = 8,000 ms, TE = 96 ms, FOV = 224 mm, 52 slices, 2 mm isotropic resolution). All b0 images were collected at the beginning of the sequence.

### Diffusion Tensor Imaging Preprocessing and Analysis

All DTI data were processed and analyzed using the Functional MRI of the Brain (FMRIB) software library (FSL v5.0.10). Raw images were pre-processed to correct for motion and residual eddy current distortion using a 12-parameter affine alignment to the corresponding b0 image *via* FSL’s eddy_correct command. A three-dimensional volume with no diffusion was generated using the nodif command, and a brain mask was generated using FMRIB’s brain extraction tool (BET v2.1) to exclude non-brain voxels from further consideration (Smith et al., 2006). Default BET settings were adjusted if brain tissue was erroneously misclassified as skull. Next, FMRIB’s Diffusion Toolbox (FDT v3.0) was used to fit the diffusion tensor and calculate FA.

Registration of FA images into MNI152 space and subsequent voxel-wise analyses followed a series of procedures known as Tract-Based Spatial Statistics (TBSS v1.2; Smith et al., 2006,^1^), as described in our previous work (Gold et al., 2010; Johnson et al., 2012). Briefly, the first step in this process was to remove likely outliers from the fitted tensor by eroding brain edge artifacts and zeroing the end slices. Second, all subjects’ FA images were aligned to the FMRIB58_FA_1 mm template using a nonlinear registration approach based on free-form deformations and B-Splines (Rueckert et al., 1999). FA datasets were then affine registered and resampled to 1 × 1 × 1 mm MNI152 space. All subsequent processing was carried out in this standardized space.

All MNI-transformed FA images were then averaged to generate a mean FA image that was used to create a common WM tract skeleton. This skeleton then reached threshold at an FA value of 0.2 in order to minimize partial voluming effects after warping across subjects. Each participant’s aligned FA image was subsequently projected onto the FA skeleton, in order to account for residual misalignments between participants after the initial nonlinear registration.

Region of interest masks were created to isolate WM tracts using validated DTI templates. First, the fornix cluster was isolated from the JHU-ICBM-labels-1 mm image using fslmaths. The Fornix_FMRIB_FA1mm.nii.gz template (Brown et al., 2017) was then added to this mask to generate a more inclusive fornical ROI. Next, corpus callosum, cingulum, and internal capsule masks were generated by combining each segmental component into a single image. Each mask was then binarized using the -bin option. Finally, the ILF mask was isolated from the JHU WM Tractography Atlas using fslmaths. All masks were then multiplied by the mean FA skeleton mask in order to generate skeletonized versions of each ROI. Each mask was substituted for the mean_FA_skeleton_mask in subsequent nonparametric permutation analyses (Randomise, FSL).

A voxelwise multiple regression analysis was performed to explore potential relationships between HDL-C, LDL-C and FA. Age, sex, and body mass index (BMI) were included as covariates of no interest in all analyses. In addition, similar analyses were performed for each the cingulum, corpus callosum, ILF, and internal capsule. A voxelwise permutation nonparametric test (using 500 permutations) was employed using a threshold-free cluster enhancement (TFCE), in order to avoid the use of an arbitrary threshold in the initial cluster formation. Results then reached threshold at *P* < 0.05 (corrected for multiple comparisons).

## Results

Demographic and serum lipoprotein data are shown in Table 1. There was a significant difference in height, weight, BMI and HDL-C between sexes. Male participants were taller (*F*_(1,86)_ = 78.3, *p* < 0.0001), weighed more (*F*_(1,86)_ = 40.6, *p* < 0.0001), and had higher BMIs (*F*_(1,86)_ = 4.6, *p* = 0.034). Female participants had significantly higher serum HDL-C values (*F*_(1,86)_ = 20.1, *p* < 0.0001). LDL-C levels did not differ between sexes (*F*_(1,86)_ = 0.064, *p* = 0.801).

**Table 1:** Demographic data and lipoprotein levels.

Subjects	Age	Height (m)	Weight (kg)	HDL (mg/dL)	LDL (mg/dL)	BMI (kg/m^2^)
*n* = 87	65.5 (3.9)	1.69 (0.10)	77.3 (16.3)	64.9 (20.1)	103.9 (36.7)	26.87 (4.7)
Female *n* = 53	65.4 (4.1)	1.64 (0.07)	69.8 (12.2)	72.1 (18.9)	103.1 (34.9)	26.02 (4.3)
Male *n* = 34	65.9 (3.8)	1.78** (0.07)	88.9** (15.0)	53.6** (16.6)	105.1 (39.8)	28.20* (5.0)

Abbreviations: m, meters; kg, kilograms; HDL, high-density lipoprotein; LDL, low-density lipoprotein; mg/dL, milligrams per deciliter; BMI, body mass index. Note: values are means and values in parentheses are SD. *P < 0.05; **P < 0.0001.

**Figure 1:**
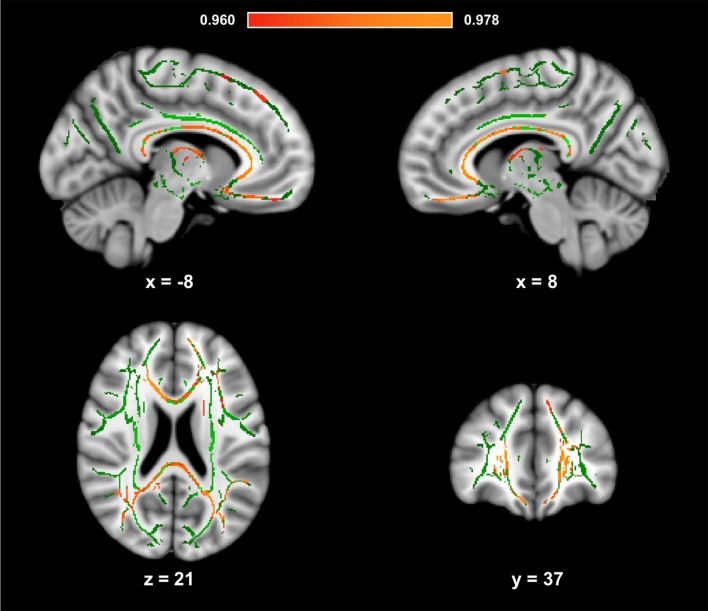
High-density lipoprotein cholesterol (HDL-C) is positively associated with fractional anisotropy (FA). Slices highlight the positive correlation observed throughout the white matter (WM) skeleton after controlling for age, sex, and body mass index (BMI; Red-Yellow). The anatomic underlay used for illustration is the MNI152 T1-weighted 1 mm brain. The registered average FA skeleton is represented in green. The numbers below each slice represent the respective, *x*, *y*, and *z* coordinates in MNI space. The scale represents the minimum and maximum P-1 values that fall above 0.95.

Figure 1 presents the results of the voxelwise analysis between HDL-C and FA. After controlling for age, sex, and BMI, a positive correlation was observed between HDL-C and diffuse regions of the WM skeleton (1-*p* = 0.980; *p* = 0.020). HDL-C did not demonstrate a negative relationship with FA (1-*p* = 0.820; *p* = 0.18).

**Figure 2:**
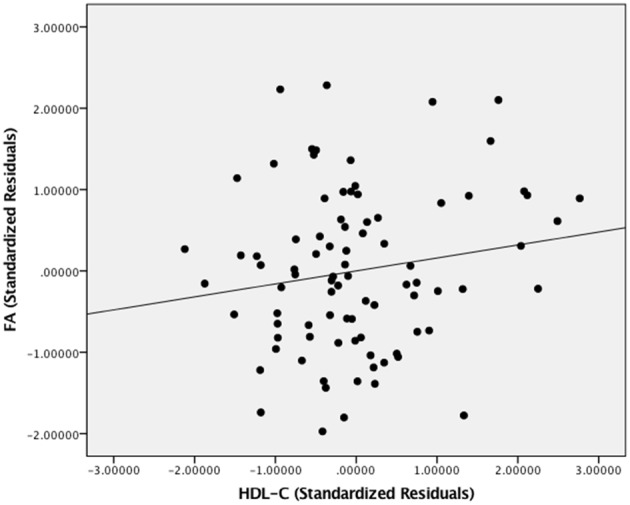
The relationship between HDL-C and FA. Scatter plot illustrating the relationship between HDL-C and WM microstructure as a result of the whole-brain voxelwise analysis. The axes represent standardized residuals of FA and HDL-C after controlling for age, sex, and BMI.

Figure 2 presents the scatter plot illustrating the relationship between HDL-C and FA. Axes represent standardized residuals after regressing the variables of interest (HDL-C and FA) onto age, sex, and BMI.

**Figure 3:**
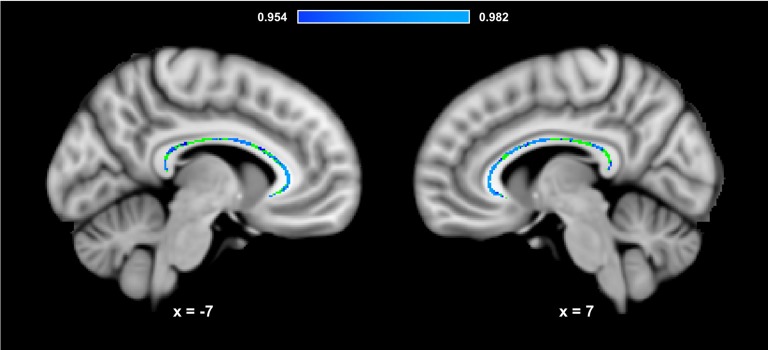
HDL-C is associated with corpus callosum WM microstructure. Slices highlight the corrected positive association observed in the corpus callosum after controlling for age, sex, and BMI (Blue-Light Blue). The anatomical is described in Figure 1. The corpus callosum skeleton mask used for the region of interest analysis is represented in green. The numbers below each slice represent the respective *x*, *y*, *z* coordinates in MNI space. The scale represents the minimum and maximum P-1 values that fall above 0.95.

**Figure 4:**
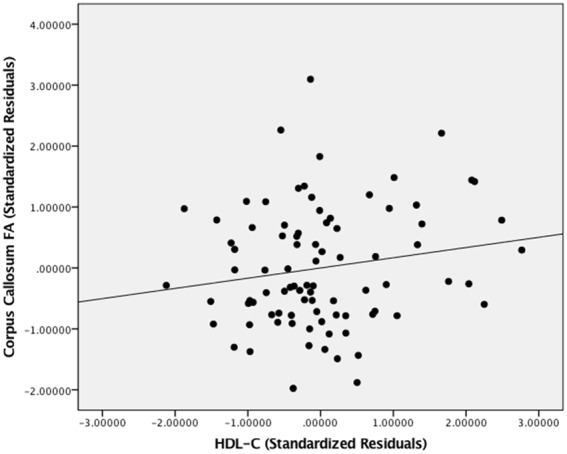
The relationship between HDL-C and FA. Scatter plot illustrating the relationship between HDL-C and WM microstructure as a result of the region of interest analysis in the corpus callosum. The axes represent standardized residuals of FA and HDL-C after controlling for age, sex, and BMI.

Figure 3 presents the results of the region of interest analyses between HDL-C and FA in the corpus callosum. After controlling for age, sex, and BMI, a positive correlation was observed between HDL-C and FA in the corpus callosum (1-*p* = 0.984; *p* = 0.016). Figure 4 presents the scatter plot illustrating the relationship between HDL-C and FA in the corpus callosum. Axes represent standardized residuals after regressing the variables of interest (HDL-C and FA) onto age, sex, and BMI. HDL-C did not demonstrate a negative relationship with FA in the corpus callosum (1-*p = 0.046;* *p* = 0.954). Further, LDL-C did not show a relationship with FA in the corpus callosum.

**Figure 5:**
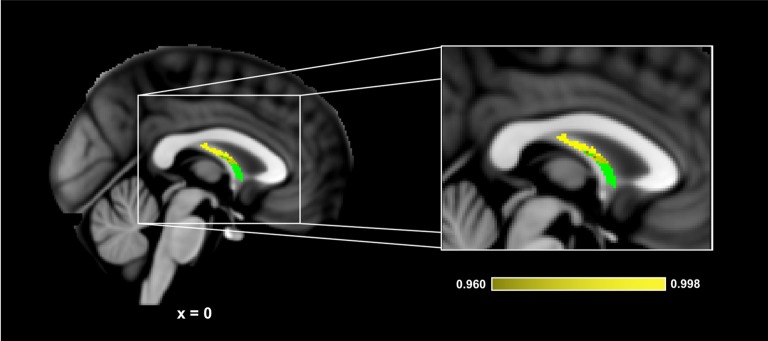
HDL-C is associated with fornix WM microstructure. Slices highlight the corrected positive association observed in the corpus callosum after controlling for age, sex, and BMI (Yellow). The anatomical is described in Figure 1. The fornix skeleton mask used for the region of interest analysis is represented in green. The numbers below each slice represent the respective *x*, *y*, *z* coordinates in MNI space. The scale represents the minimum and maximum P-1 values that fall above 0.95.

**Figure 6:**
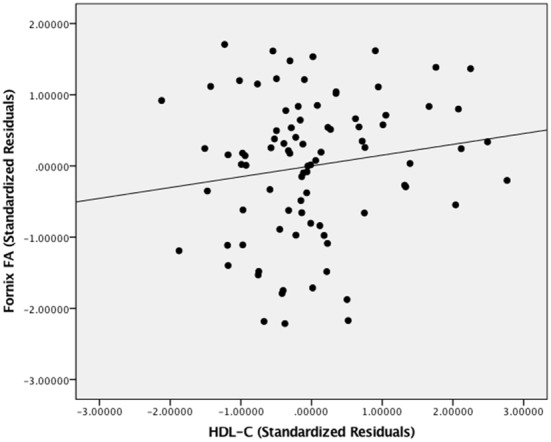
The relationship between HDL-C and FA. Scatter plot illustrating the relationship between HDL-C and WM microstructure as a result of the region of interest analysis in the fornix. The axes represent standardized residuals of FA and HDL-C after controlling for age, sex, and BMI.

Figure 5 presents the results of the region of interest analyses between HDL-C and FA in the fornix. After controlling for age, sex, and BMI, a positive correlation was observed between HDL-C and FA in the fornix (1-*p* = 0.998; *p* = 0.002). Figure 6 presents the scatter plot illustrating the relationship between HDL-C and FA in the fornix. HDL-C did not demonstrate a negative relationship with FA in the fornix (1-*p* = 0.060; *p* = 0.940). Further, LDL-C did not show a relationship with FA in the fornix.

No relationship was observed between HDL-C and WM microstructure in the internal capsule (positive, 1-*p* = 0.812; *p* = 0.188; negative, 1-*p* = 0.460; *p* = 0.540) or ILF (positive, 1-*p = 0.908*; *p* = 0.092; negative, 1-*p* = 0.018; *p* = 0.982).

## Discussion

The present study represents the first exploration of the relationship between non-fasting HDL-C, LDL-C, and WM microstructure in a homogeneous sample of community-dwelling older adults. Our results build upon findings that HDL-C may help to maintain the structural integrity of the aged brain (Ward et al., 2010; de Bruijn et al., 2014). Specifically, we found that HDL-C was associated with WM microstructure (FA) in diffuse regions of the brain. We also observed a significant relationship between HDL-C and WM microstructure in the corpus callosum and fornix. The implications of these findings are discussed below.

Advancing age is associated with deleterious changes in brain structure. This study focused on determining the relationship between a modifiable lifestyle variable, HDL-C, and a neuroimaging marker of WM microstructure, FA. We observed a positive association between HDL-C and FA across a diffuse portion of the brain’s WM. However, we did not observe a relationship between LDL-C and WM microstructure. This supports previous findings that adolescent LDL-C was not associated with FA in young adulthood (Warstadt et al., 2014). Further, it has been proposed that non-fasting levels of HDL-C may be more diagnostically accurate for the assessment of cardiovascular risk than non-fasting levels of LDL-C (Fatima et al., 2016).

We also observed a significant relationship between HDL-C and FA in the corpus callosum and fornix. These HDL-C-FA relationships are of particular interest due to the role the corpus callosum and fornix play in maintaining cognitive function in healthy adults (Fletcher et al., 2013; Douet and Chang, 2015; Huang et al., 2015; Zhang et al., 2015). Further, corpus callosum and fornix microstructure are associated with AD and AD pathology (Ardekani et al., 2014; Bachman et al., 2014; Gold et al., 2014; Kantarci et al., 2017). For example, Gold et al. (2014) observed a positive correlation between CSF measures of Aβ and fornix WM microstructure, and Zhang et al. (2015) observed that APOε4 carriers without cognitive decline demonstrated inverse relationships between cognition and WM microstructure in the corpus callosum and fornix. Thus, findings from this study suggest that higher levels of HDL-C may help to preserve WM microstructure in tracts associated with cognition in late adulthood.

The age-related homogeneity of our cohort builds on previously reported relationships between HDL-C and WM in young adults and in cognitively healthy mixtures of middle-aged and older adults (Williams et al., 2013; Warstadt et al., 2014; Ryu et al., 2017). For example, Warstadt et al. (2014) observed that adolescent levels of non-fasting HDL-C were associated with WM microstructure in young adulthood. However, fasting HDL-C values have shown positive and negative relationships in mixtures of middle-aged and older adults using a whole brain approach (Williams et al., 2013). Middle-aged and older adult samples have also demonstrated that poorer health is associated with reduced WM microstructure (Power et al., 2017).

The positive association observed between HDL-C and WM microstructure across diffuse regions of the brain may help to explain the positive relationship between high levels of HDL-C and maintained cognition in adults with exceptional longevity (Atzmon et al., 2002; Barzilai et al., 2006), including centenarians. Further, low levels of HDL-C are associated with cognitive impairment, including poor memory, in middle-aged and older adult cohorts (van Exel et al., 2002; Singh-Manoux et al., 2008; Song et al., 2012). Low levels of HDL-C are also associated with reduced GM and WM health (Crisby et al., 2010; Ward et al., 2010). Thus, this modifiable lifestyle variable may support cognitive function via preserved structure, but future studies are warranted to confirm causal relationships.

Several mechanisms offer insight into the potential physiology behind our findings. HDL-C is atheroprotective and may help to preserve WM health *via* vascular mechanisms. Many of the atheroprotective benefits of HDL-C target the endothelium. For example, HDL-C removes cholesterol from arterial walls (von Eckardstein et al., 2001), increases endothelial nitric oxide (NO) synthase activity (Drew et al., 2004), and induces vasorelaxation (Nofer et al., 2004). Superior endothelial health is associated with better WM microstructure (Johnson et al., 2017). In addition to the efflux of cholesterol in arterial walls, HDL-C also contributes to the efflux of cholesterol in neurons (Demeester et al., 2000). Another potential mechanism is related to the ability of sub-fractions of HDL-C to bind Aβ in CSF (Koudinov et al., 2001). The cross-linking of HDL-C and Aβ in CSF may limit the deposition of the Aβ in the brain. Our findings contribute to the proposed neuroprotective attributes of a modifiable lifestyle variable such as HDL-C.

However, non-modifiable variables, such as APOEε4 status and sex, can also influence the relationship between cholesterol and neuroimaging measures of WM (Willey et al., 2014; Cavedo et al., 2017; Yin et al., 2018). For example, Willey et al. (2014) examined the association between lipid profiles and WM hyperintensity volumes in a population-based cohort over the age of 55. Findings demonstrate that greater WM hyperintensity volume was associated with worsening of HDL-C over time, and that APOEε4 carriers with total cholesterol >200 mg/dL had a trend towards smaller WM hyperintensity volumes than those with total cholesterol values <200 mg/dL. Yin et al. (2018) also observed an interaction between HDL-C and sex, such that HDL-C was inversely associated with WM lesions in females but not males. This study controlled for sex differences but did not collect data associated with APOEε4 status.

The present study has several caveats that highlight areas that need further investigation. First, the cross-sectional nature of our study limits the ability to draw causal conclusions about HDL-C and WM microstructure. The relationship observed in the present study serves to justify future longitudinal designs to determine if improved HDL-C preserves WM microstructure. Further, such longitudinal studies should determine any cognitive benefits associated with HDL-C and WM microstructure. Second, we did not control for APOEε4 carrier status. As previously referenced, the relationship between APOEε4 status, cholesterol, and WM health is complex (Willey et al., 2014; Cavedo et al., 2017). Third, the relationship between HDL-C and brain health can depend on a myriad of factors (Hottman et al., 2014; Kontush, 2014; Koch and Jensen, 2016; Power et al., 2017). Future studies should consider lipoprotein genetic interactions, fasting levels of other serum lipoproteins, race, and CSF biomarkers. For example, Aβ is related to fornix WM microstructure (Gold et al., 2014), and accumulation can lead to myelin breakdown (Xu et al., 2001).

In conclusion, our results demonstrate that non-fasting HDL-C is positively correlated with WM microstructure in diffuse regions of the brain and in WM regions demonstrating inverse relationships with cognition in late adulthood. The observed HDL-C-WM relationships were observed after controlling for age, sex, and BMI, and highlight the relationship between WM microstructure and modifiable lifestyle variables. In addition, the novel examination of the relationship between non-fasting HDL-C and WM microstructure allows for a more accurate characterization of how circulating lipid levels influence the structural integrity of the brain in late adulthood. These findings motivate future longitudinal studies aimed to determine if improving atheroprotective biomarkers, through lifestyle modification, attenuates age-related declines in WM microstructure.

## Ethics Statement

Informed consent was obtained from each participant under an approved University of Kentucky Institutional Review Board protocol.

## Author Contributions

NJ: all aspects of the data collection, analysis, and manuscript development. DR: data collection and analysis. AB: data analysis and manuscript preparation. JC: data collection and manuscript preparation. VG, SL, DP and BG: data collection and analysis, manuscript preparation.

## Conflict of Interest Statement

The authors declare that the research was conducted in the absence of any commercial or financial relationships that could be construed as a potential conflict of interest.

## Footnotes

^1^http://www.fmrib.ox.ac.uk/fsl/tbss/
